# Experimental Assessment and Numerical Modeling of the Bond–Slip Correlation for Steel Rebars in r.c. Members

**DOI:** 10.3390/ma15030951

**Published:** 2022-01-26

**Authors:** Pietro Croce, Paolo Formichi, Filippo Landi

**Affiliations:** Department of Civil and Industrial Engineering, University of Pisa, Largo Lucio Lazzarino 1, 56122 Pisa, Italy; p.formichi@ing.unipi.it (P.F.); filippo.landi@ing.unipi.it (F.L.)

**Keywords:** bond–slip, reinforced concrete, non-linear behavior, numerical analysis, crack pattern, crack opening, bond stress

## Abstract

Refined non-linear static or dynamic analyses are increasingly used to assess the behavior of new and existing reinforced concrete structures. To perform these analyses, an adequate knowledge of the force–displacement, bending moment–curvature, and bending moment–rotation curves of relevant parts of structural members is needed, and modeling the bond–slip correlation for steel rebars becomes fundamental. The paper presents the results of an experimental campaign on r.c. specimens under tension, aiming, differently from previous studies, to better reproduce the bond–slip relationship accounting for the local confinement and anchorage conditions of real structural members. Resorting to an original numerical procedure allowing us to predict the relative displacement between steel reinforcement and the surrounding concrete in a reinforced concrete element, once assigned the stress in the naked steel bar and the bond–slip law, the experimental results are compared with the numerical outcomes obtained by adopting codified bond–slip laws. The comparison highlights that experimental values of sliding are well below those that are commonly given in existing bond slip laws, such as that adopted by the CEB-FIP Model Code. A new bond–slip model, which is able to satisfactorily predict actual strain fields and slips along the investigated r.c. elements, is thus proposed with the final aim of extending its implementation into non-linear analyses of r.c. structures.

## 1. Introduction

Non-linear static or dynamic analyses are increasingly used to study the behavior of reinforced concrete structures, especially for seismic designs [1] as well as for the structural assessments of existing buildings [2,3]. Carrying out non-linear analysis requires knowledge of the actual force–displacement, bending moment–curvature, and bending moment–rotation curves of relevant parts of structural members [4,5,6,7]. These capacity curves are needed for the evaluation of the available ductility of structural elements as well as for an appropriate estimation of the relevant structural parameters, which are required to perform equivalent elastic analyses, widely used as a simplified approach. Evidently, the curves can be obtained experimentally, through sophisticated and typically expensive full-scale ad hoc tests, or numerically, by resorting to refined theoretical models that have been suitably validated [8].

The non-linear force–displacement or bending moment–rotation relationships of r.c. members depend on the crack widths and on the crack pattern, and on the slip between concrete and reinforcing steel. Advanced models should appropriately consider all these local aspects as key issues for a suitable model [9,10,11,12,13,14], which are generally ignored in classical simplified approaches. Owing to the increasing interest in the assessment of existing r.c. structures [15,16], refined structural analyses, allowing us to reduce the impact on the structural strengthening interventions of safe sided assumptions often adopted in simplified approaches, are getting extensively used. 

Moreover, experimental studies [17,18] have demonstrated that the classical approach to the analysis of reinforced concrete structures, based on the assumption of a perfect bond between steel rebars and concrete, is unsuitable for predicting the actual displacements/rotations of structural members, leading to remarkable overestimates in the evaluation of elements’ stiffness. For these reasons, several studies [19,20,21] have been devoted to improving the approach, to allow a more refined modeling of the behavior of cracked r.c. members, based on specific bond–slip correlations.

In this paper, the results of an experimental campaign carried out on r.c. ties at the University of Pisa are presented and discussed. The experimental campaign and the type of test specimen represent an evolution of previous experimental studies [22,23,24] and have aimed at simulating the behavior of a real r.c. member under tension.

The test results, in terms of steel strains and the derived slips and bond stresses, are compared with the corresponding theoretical data, which were obtained by implementing a numerical procedure for the solution of the differential equations governing the interaction between steel bars and concrete [25]. The numerical procedure, described in Section 2, allows us to predict the relative displacement between the steel reinforcement and the surrounding concrete in a reinforced concrete tie, once we have assigned the stress in the naked steel bar and the bond–slip law. In fact, the definition of the bond–slip model is a crucial aspect, which has been the subject of numerous studies, leading to the model initially proposed in [9] and then adopted in the CEB-FIP Model Code [26,27].

The bond–slip model provided in the CEB-FIP Model Code, based on the full confinement of concrete members and often adopted in current practice, was adopted in the numerical analyses as a reference model to simulate the results obtained from tests. The comparison showed significant discrepancies from the experimental results, thus indicating the need for a new bond–slip model to predict the behavior of real r.c. members. 

In this paper, an alternative effective bond–slip model is proposed with the aim of replicating experimental results and providing a suitable model to be used for the refined structural analyses of real reinforced concrete structures. 

## 2. Bond–Slip Modeling in r.c. Structures

### 2.1. Analytical Modeling

**Figure 1:**
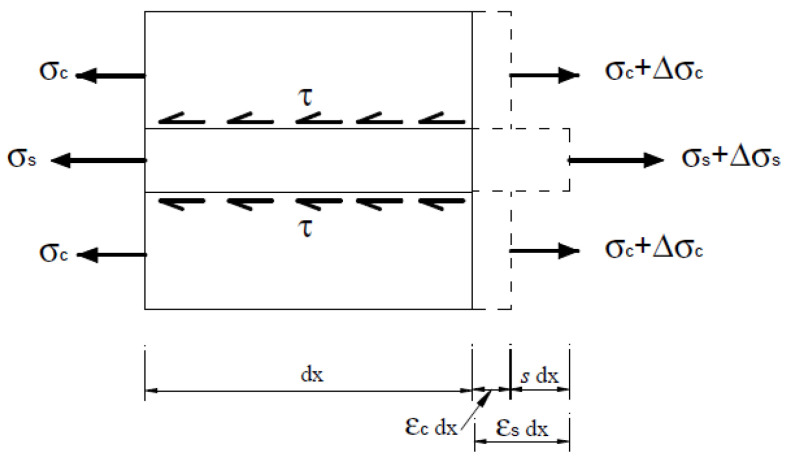
Stresses and elongations on an infinitesimal r.c. tie.

The classical equations governing the bond–slip problem can be derived considering the equilibrium and compatibility conditions of an infinitesimal r.c. tie (see Figure 1). Considering a tie, the length of which is *dx*, made up by one centered rebar and by the surrounding portion of concrete in tension, subject to a tensile force *F*, the equation governing the global equilibrium of the tie is trivially

(1) $$F=F_{s}+F_{c}$$

where $F_{s}$ and $F_{c}$ are the force acting on steel and on concrete, respectively. Differentiating Equation (1) gives

(2) $$dF=dF_{s}+dF_{c}=d\sigma_{s}\frac{\pi \Phi^{2}}{4}+A_{c}\left(x\right)d\sigma_{c}+\sigma_{c}dA_{c}\left(x\right)$$

and then

(3) $$d\sigma_{c}=-\rho \left(x\right)d\sigma_{s}-\sigma_{c}\frac{dA_{c}\left(x\right)}{A_{c}\left(x\right)}$$

where Φ is the diameter of the steel bar, $A_{c}\left(x\right)$ is the concrete area affected by the bond stress diffusion, $\rho \left(x\right)$ is the geometric reinforcement ratio, and $\sigma_{s}$ and $\sigma_{c}$ are the stresses in steel and concrete, respectively. 

Considering the bond stress $\tau \left(s\right)$ and the function of the slip s, the local equilibrium of the rebar over the length dx is given by

(4) $$d\sigma_{s}\frac{\pi \Phi^{2}}{4}=\pi \Phi \tau \left(s\right)dx$$

and for the compatibility condition,

(5) $$\left(\varepsilon_{s}-\varepsilon_{c}\right)dx=ds$$

it yields

(6) $$\frac{ds\left(x\right)}{dx}=\varepsilon_{s}-\varepsilon_{c},$$

with $\varepsilon_{s}$ and $\varepsilon_{c}$ being the strains in steel and concrete, respectively.

Combining Equations (2), (4) and (6), the bond–slip problem can be described by the following differential equation

(7) $$\frac{d^{2}}{dx^{2}}s=\frac{4\tau \left(s\right)}{\Phi }\left(\frac{d}{d\sigma_{s}}\varepsilon_{s}+\rho \left(x\right)\frac{d}{d\sigma_{c}}\varepsilon_{c}\right)+\frac{\sigma_{c}}{A_{c}\left(x\right)}\frac{d}{dx}A_{c}\left(x\right)$$

which can be solved numerically, under appropriate boundary conditions, once the stress–strain relationships for steel and for concrete under tension, the bond–slip law, and the dependence of $A_{c}$ on x are defined.

### 2.2. Numerical Modeling

**Figure 2:**
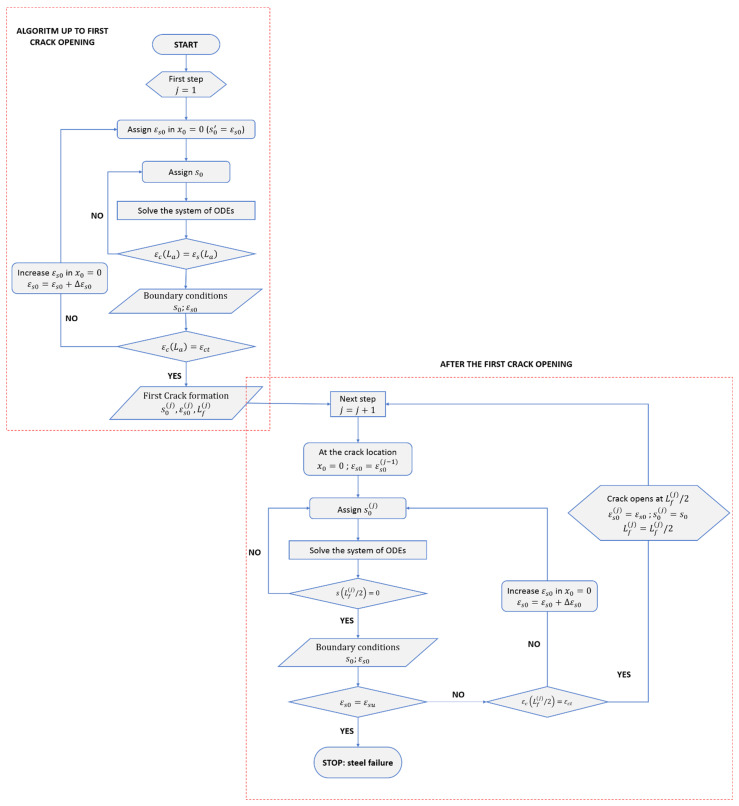
Flowchart of the numerical procedure to solve the bond–slip problem in r.c. elements.

A numerical procedure to solve the non-linear differential equation that governs the bond–slip problem was proposed in [25]. The algorithm is summarized in Figure 2 and is based on an iterative shooting solution of the system of ODEs

(8) $$\left\{\begin{array}{c} \frac{d}{dx}s=\varepsilon_{s}\left(\sigma_{s}\right)-\varepsilon_{c}\left(\frac{F-\sigma_{s}A_{s}}{A_{c}\left(x\right)}\right)\\ \frac{d}{dx}\sigma_{s}=\frac{4}{\Phi }\tau \left(s,x\right) \end{array}\right.$$

which can be suitably derived from Equation (7).

The first phase of the algorithm allows us to define the boundary conditions up to the first crack opening in a reinforced concrete tie of length L by means of the following steps:

1. the origin of the *x*-axis is set at the starting cross-section of the tie, $x=x_{0}=0$, where $\sigma_{c}=0$, and half of the tie is subdivided into N intervals of constant length Δx;
2. the constitutive laws σ−ε for steel and concrete under tensile stresses are assigned, as well as the bond–slip law τ−s and the $A_{c}\left(x\right)$ function;
3. tentative boundary conditions, $s_{0}$ and $\varepsilon_{s0}$ in x=0, are assigned, and the bond and tensile stress, $\tau_{0}\left(s_{0}\right)$ and $\sigma_{s0}\left(\varepsilon_{s0}\right)$, are evaluated using the previously defined bond–slip law and the steel constitutive law;
4. the system of Equation (8) is integrated in the *i*-th interval using the Runge–Kutta 4th order method [28], determining the slip $s_{i}$ and the steel stress $\sigma_{s,i}$ at the end of the interval $x_{i}$;
5. the bond stress, $\tau_{i}\left(s_{i}\right)$, in $x_{i}$ is evaluated using the bond–slip law;
6. the stress in the concrete section, $\sigma_{c,i}$, is calculated through the equilibrium condition
  (9) $$\sigma_{c.i}=\frac{\left(F-\sigma_{s,i}A_{s}\right)}{A_{c}\left(x_{i}\right)}$$
7. the iteration is stopped when $s_{i}=0$, and consequently $\tau_{i}=0$, or when i=N, and convergence is checked (see step 9);
8. the process is iterated from step 4 for the next interval i=i+1;
9. the convergence is checked—if the concrete strain can be considered equal to the steel strain for the assigned tolerance, $\varepsilon_{c.i}=\varepsilon_{s.i}$ in $x_{i}$, convergence is achieved, but otherwise, a new value is assigned to $s_{0}$, keeping the value of $\varepsilon_{s0}$ unchanged, and the process is iterated from step 3 till convergence. The search for $s_{0}$ is performed using one of the numerical root-finding methods, such as the secant method or the Newton–Raphson method;
10. once the convergence is achieved, the actual value of $s_{0}\left(\varepsilon_{s0}\right)$ is retained together with the actual transfer length $L_{a}\left(\varepsilon_{s0}\right).$

By means of the presented steps, the boundary conditions at x=0, $s_{0}\left(\varepsilon_{s0}\right)$ and $\varepsilon_{s0}$, can be found for each stress level in the naked bar, $\sigma_{s0}\left(\varepsilon_{s0}\right)$. By increasing the value of $\varepsilon_{s0}$, the loading process is simulated until the ultimate tensile strain in the concrete, $\varepsilon_{ct}$, is attained at a distance $L_{a}$ from the end sections, such as $\varepsilon_{c}\left(L_{a}\right)=\varepsilon_{ct}$, and the first crack opens at $x=L_{f}^{\left(j=1\right)}\geq L_{a}$. Actually, since the concrete tensile strain in each section belonging to the interval $L_{a}\leq x\leq L-L_{a}$ satisfies the condition $\varepsilon_{c}=\varepsilon_{ct}$, the location of the first crack is a random section of that interval, but, for the sake of the numerical algorithm, considering that the number of cracks and their distances in the final (stabilized) crack pattern are quite independent of the order in which subsequent cracks appear, the first crack is assumed to occur in the central zone of the tie.

After the first crack has opened, the numerical procedure is suitably modified to evaluate the opening of new cracks. At each step *j*, the portion of the tie of length $L_{f}^{\left(j-1\right)}$ is considered, and the following operations are carried out:

11. the origin of the *x*-axis is set at the starting cross-section of the considered portion of the tie, bounded by adjacent cracks spaced $L_{f}^{\left(j-1\right)}$, and the half of the portion is subdivided into M intervals of constant length Δx;
12. the boundary conditions $s_{0}$ and $\varepsilon_{s0}$ in x=0 are assigned following from the previous step, i.e., after the $\left(j-1\right)$-th crack formation phase, leading to $\varepsilon_{s0}=\varepsilon_{s0}^{\left(j-1\right)}$, and the bond stress and tensile stress are evaluated accordingly;
13. the system of ODEs in Equation (8) is solved by repeating the same procedure (steps 3 to 9) and checking convergence, which is achieved when slip s vanishes at the midpoint of the tie, i.e., $s\left(L_{f}^{\left(j-1\right)}/2\right)=0$;
14. if $\varepsilon_{c}(L_{f}^{\left(j-1\right)}/2)$ has reached the concrete ultimate tensile strain $\varepsilon_{ct}$, a new crack opens at $L_{f}^{\left(j-1\right)}/2$, otherwise the value of $\varepsilon_{s0}$ is increased and step 13 is repeated till the condition $\varepsilon_{c}(L_{f}^{\left(j-1\right)}/2)=\varepsilon_{ct}$ is satisfied. Once new crack opens, the new portion of the tie is set to $L_{f}^{\left(j\right)}=L_{f}^{\left(j-1\right)}/2$ and the process from 11 is iterated until the steel strain $\varepsilon_{s0}$ reaches the ultimate value $\varepsilon_{su}$.

Once one has completed the procedure, the width of each crack is evaluated by summing the quotas of $s_{0}$ pertaining to the two parts of the tie delimited by the crack. 

**Figure 3:**
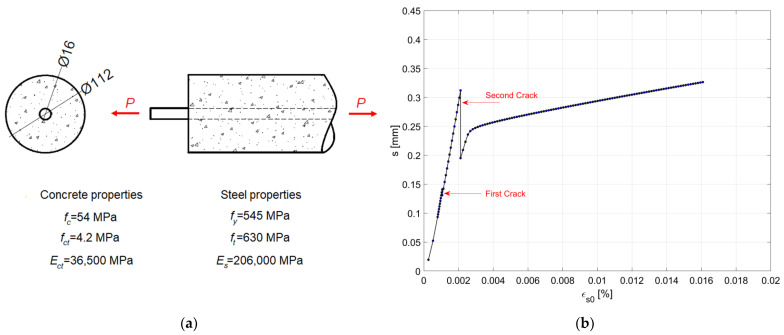
Example of the implementation of the procedure: (**a**) reinforced concrete cylindrical tie under axial load P and experimental materials properties (unit: mm); (**b**) resulting slip s-strain $\varepsilon_{s0}$ diagram considering the CEB bond–slip model.

An example of an application of the procedure is shown in Figure 3b, considering the reinforced concrete tie illustrated in Figure 3a, where the material mechanical properties (concrete compressive strength $f_{c}$, concrete tensile strength $f_{ct}$, concrete elastic modulus $E_{ct}$, steel yielding strength $f_{y}$, steel ultimate tensile strength $f_{t}$, and steel elastic modulus $E_{s}$) are also indicated. The r.c. cylindrical tie is characterized by a diameter Φ=112 mm, length l=0.8 m, and by a 16 mm B450C steel rebar located in the center. In the analysis, Ramberg–Osgood constitutive laws [29] are assumed for the steel and concrete tensile behavior and the CEB model [26] is adopted for the bond–slip law. The example shows the ability of the algorithm to efficiently predict the cracking of the r.c. tie.

## 3. Experimental Results

An experimental test campaign was carried out to evaluate the bond–slip correlation for steel rebars in r.c. members. The innovative experimental technique is based on the study of the behavior of long specimens, where rebars are directly instrumented with strain gauges placed on their external surface.

The instrumentation of rebars with strain gauges is a relevant technical issue in the analysis of bond–slip behavior, it being necessary to directly measure the steel stresses at many points along the rebar, closely spaced from each other, allowing the effective placement of the electrical connections and not altering the stress transfer at the rebar surface. Examples of strain gauge arrangements can be found in the literature [22].

**Figure 4:**
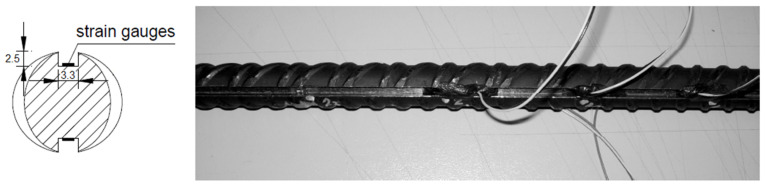
Insertion of electrical strain gauges in the ribbed steel rebar (unit: mm).

The original instrumentation of the steel bars adopted here is obtained by milling the ribbed rebar along two diametrically opposite generators, matching the smooth parts of its external surface (see Figure 4). In this way, the milled section, which is large enough to insert strain gauges with a 3 mm grid length, does not significantly alter the external surface of the bar, and does not modify the ribbed part. Strain gauges are glued into the groove with cyanoacrylate and duly protected and waterproofed so as not to be damaged during the pouring of concrete.

The strain gauge connection wires were kept outside the bars and placed radially to the concrete cylinder, which was poured around the bar. The presence of the wires in the concrete mass introduces discontinuities into it, which, however, consist of radial breaks, alternating from the two sides of the bar, along a diametrical plane of the cylinder itself, and do not significantly alter the formation of transverse cracks and of longitudinal ones at a later stage, as was observed during tests.

The arrangement of the strain gauges’ wires and the limitation of the milled groove in the bars make it possible to use this technique and this instrumentation even in bars with a lower diameter than those used in the past [22,23]. The cross-section reduction is about 17 mm^2^, which represents just over 8% of the resistant section for a 16 mm diameter bar, which is, in any case, considered in the analysis. The reduction in the adherent section on the lateral surface of the bar is equal to 3.3 × 2 = 6.6 mm on a nominal perimeter, while that for the same bar φ16 is about 50 mm; the reduction is therefore of the order of 13%. In any case, it does not particularly affect the bond; in fact, the stress transfer is assured by the ribs, which are not affected by the milling of the groove, as clearly results from Figure 4.

The milling is carried out with low-speed milling cutters in a cooling bath to limit the thermally induced stresses generated by the preparation of the specimens.

Inside the two longitudinal millings, the electrical strain gauges were placed at 25 mm intervals, in alternate positions, so that on the same side of the bar there would be a strain gauge every 50 mm, thus reducing the local disturbance effects generated by the presence of the connecting wires.

According to the described technique, bars with lengths of their instrumented parts equal to 100 and 23 cm were prepared. Two diameters were used for the steel rebars: φ16 and φ20. The diameter of the confining concrete cylinder was assumed constant and equal to 132 mm, which corresponds to approximately 6–8 times the diameter of the embedded rebars.

**Figure 5:**
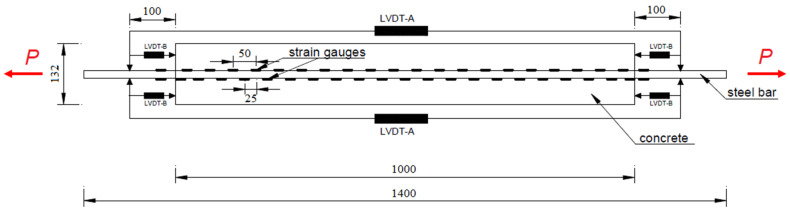
Layout of the test specimen *L* = 1000 mm under axial load P (unit: mm).

As illustrated in Figure 5, the two ends of the bar are extended beyond the concrete cylinder to allow for clamping into a universal tensile testing machine. 

In addition to strain gauges, LVDT transducers were used to measure the total elongation of the bar (LVDT A) and the slip at the two end faces of the concrete cylinder (LVDT B).

Although both specimens, the 100 cm- and 23 cm-long ones, can be classified as “long” specimens characterized by a long member behavior [25], the formation of multiple cracks is possible, allowing us to study the evolution of progressive cracking phenomena until their complete stabilization. The shorter specimens, whose length was calibrated to avoid intermediate cracks, were used to verify the evolution of the bond slip law in the absence of transversal cracking.

In Table 1, the main characteristics of the six specimens tested during the experimental campaign are illustrated together with the number of instruments used for each test.

**Figure 6:**
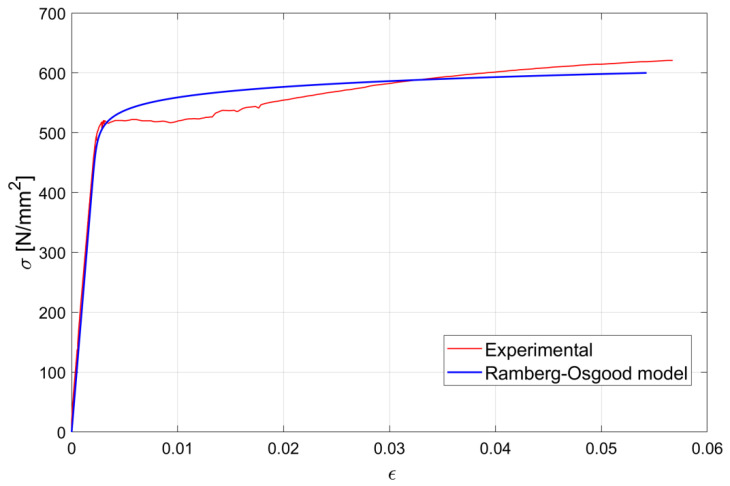
Experimental stress–strain diagram for a φ16 steel rebar used in the test campaign.

The mechanical behaviors of the materials used for each specimen were tested in the laboratory. Steel B450C rebars from the same casting were used for each specimen and mechanical properties were evaluated by means of preliminary tensile tests, conducted on specimens instrumented with a pair of strain gauges, glued in diametrical positions at the mid-section of the specimen. Figure 6 shows one of the experimental stress–strain diagrams obtained during the experimental campaign together with the analytical model obtained by adopting the Ramberg–Osgood constitutive law. Prior to concrete pouring, all the instrumented bars were tested under loads well below their elastic limit to verify the correct functioning of the installed strain gauges.

**Figure 7:**
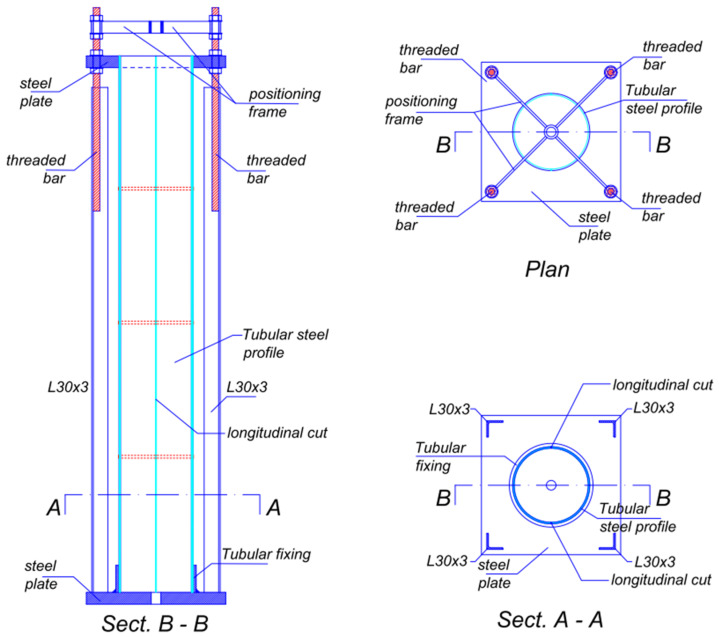
Special formworks used to prepare the cylindrical specimens.

Special formworks, consisting of two portions of a tubular steel profile with an internal diameter of 132 mm, were used to prepare the cylindrical specimen. The formworks, shown in Figure 7, were cut along the two generatrixes of a diametrical plane, to allow the arrangement of strain gauge connection wires along the line of conjunction of the two halves. The formworks were completed by a support system, which allowed us at the same time to center the bar along the axis of the cylinder, and to keep it in position during the subsequent phases of pouring and curing. Self-compacting concrete (SCC) was used to avoid any need for the compaction of castings that could potentially damage the instruments. The SCC mix was made from 515 kg/m^3^ of hyper fluid expanding cementitious binder, 200 kg/m^3^ of gravel, 600 kg/m^3^ of fine gravel, 815 kg/m^3^ of sand and 240 kg/m^3^ of water, so that the water to cement ratio was about 0.466.

During each casting, two cylindrical concrete samples were taken (except for sample n.1, for which four cylinders were taken) with a height to diameter ratio of 2, to perform compression tests and indirect (“Brazilian”) tensile tests. Compression and indirect tensile tests on concrete specimens were performed immediately before the test on each tie, and after the 28th day of concrete curing. The results are summarized in Table 2 and Table 3, where R indicates the maximum axial load sustained by the specimen.

**Figure 8:**
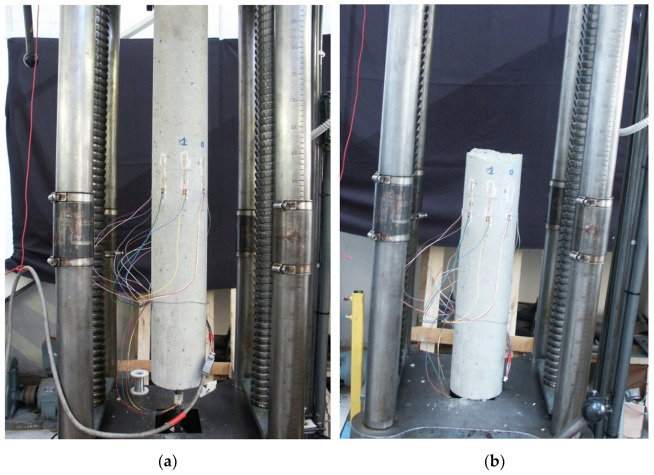
Instrumented cylindrical r.c. tie used for direct tensile tests: (**a**) at the beginning of the test; (**b**) after failure.

In addition to compression and indirect tensile tests on specimens taken during casting, special specimens were prepared for some ties to directly characterize the tensile stress–strain diagram. These specimens are similar to the “long” r.c. specimens used for the tensile tests, except for the steel bar, which is interrupted at about 25 cm (≈2$\Phi_{c}$) across the middle section of the cylinder, and has special ribs welded to the ends of the bar embedded in the concrete. In this way, the concrete section in the middle of the cylinder is subjected to a stress state that can be reasonably assumed to be uniform uniaxial tensile. On the external surface of the cylinder, eight electrical strain gauges for the concrete with an 8 cm-long base were placed in order to record the tensile deformations up to the tensile failure of the concrete. An example of the cylindrical r.c. tie used for the direct tensile test at the beginning of testing is illustrated in Figure 8a, where one can easily distinguish the strain gauges, while Figure 8b shows the tested specimen after the failure.

**Figure 9:**
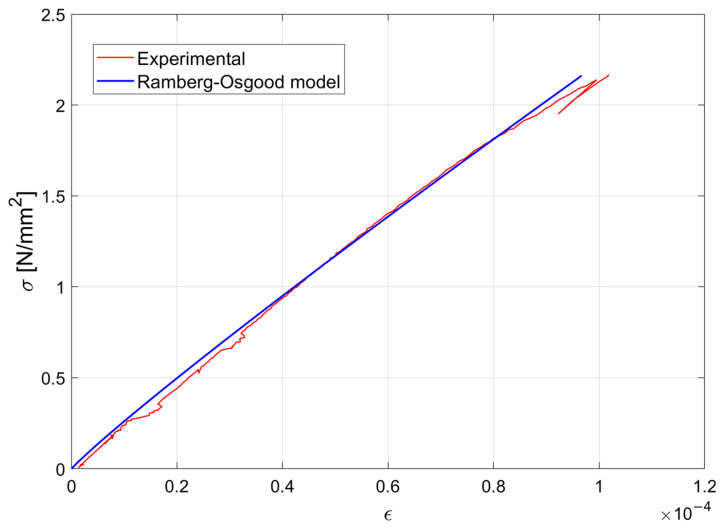
Experimental tensile stress–strain diagram for concrete (specimen n.2 in Table 4).

In Figure 9, the experimental stress–strain diagram (in red) obtained for one specimen is compared with the Ramberg–Osgood constitutive law (in blue) after calibration of the parameters.

In Table 4, the results of the tests in terms of tensile strength $f_{ct}$ and elastic modulus $E_{ct}$ are summarized and compared with the values obtained according to the formulations given in Eurocode 2 [30], depending on the compressive strength

(10) $$f_{ctm}=0.3{\left(f_{cm}-8\right)}^{\frac{2}{3}}$$

and

(11) $$E_{cm}=22,000{\left(\frac{f_{cm}}{10}\right)}^{0.3}$$

The tensile strength determined according to EN formulations is in good agreement with experimental values obtained by the indirect tensile tests, while the experimental values of the elastic modulus are generally lower than those obtained by the EN provisions. A ratio of about 0.75 is obtained between the experimental elastic modulus and that obtained according to Equation (11).

Once we had characterized the mechanical behavior of the concrete and steel rebars used for assembling r.c. specimens, the tensile tests were carried out on the six r.c. ties listed in Table 1. Tests were carried out while controlling the applied load, with an increase rate of about 5 kN/min, in conformity with the loading rate usually adopted in standard laboratory tests. In some cases, to check the slopes of the unloading and reloading cycles and their compliance with the initial slopes, an intermediate unloading cycle was performed, followed by a new reloading, always at constant speed.

### 3.1. Long Specimen

**Figure 10:**
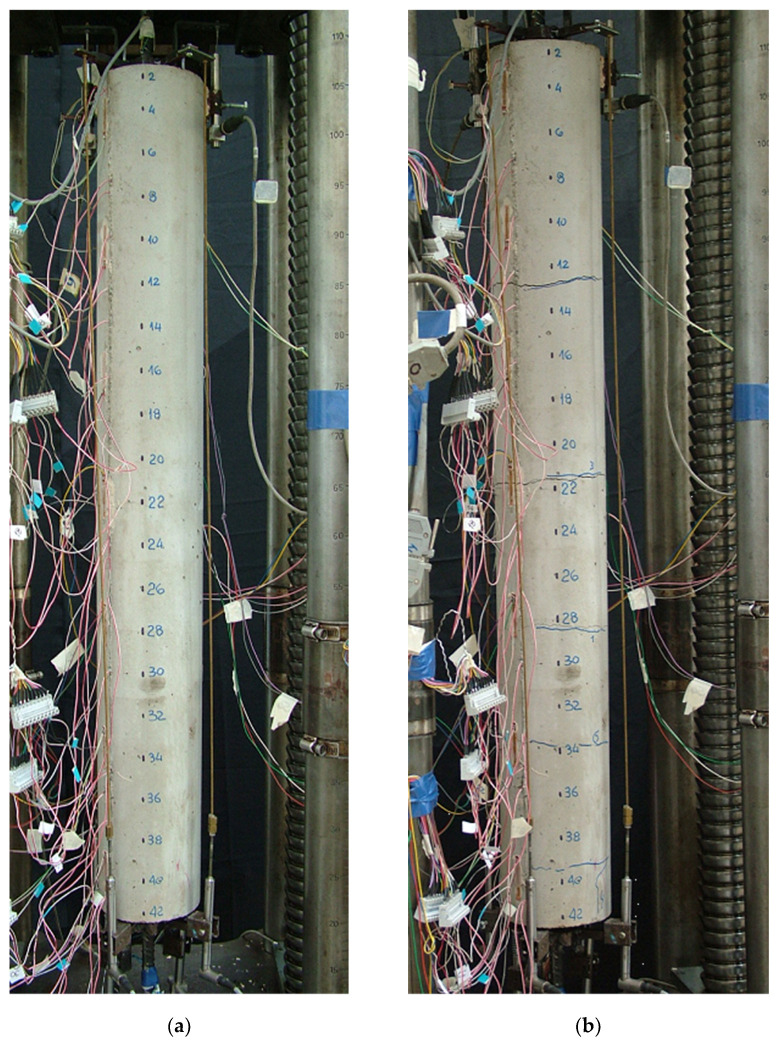
Reinforced concrete specimen n.1 tested at the Laboratory of the University of Pisa: (**a**) specimen before the test, (**b**) cracked specimen after the test.

In Figure 10, an example of a “long” test specimen (n.1 in Table 1) is shown before the test (Figure 10a) and at the end of the test (Figure 10b). In the latter, the transversal cracks, which occurred at increasing values of the load during the test, are clearly visible. 

**Figure 11:**
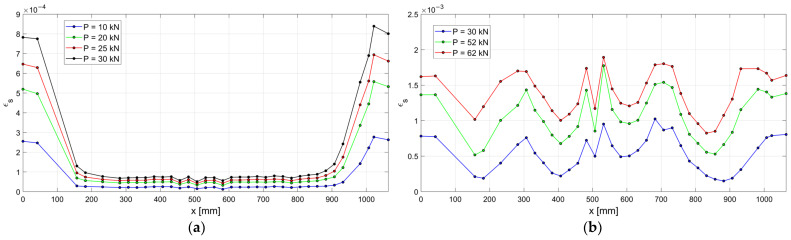
Experimental ε-x curves of the n.1 r.c. tie: (**a**) *P* < 30 kN, (**b**) 30 kN ≤ *P* ≤ 60 kN.

**Figure 12:**
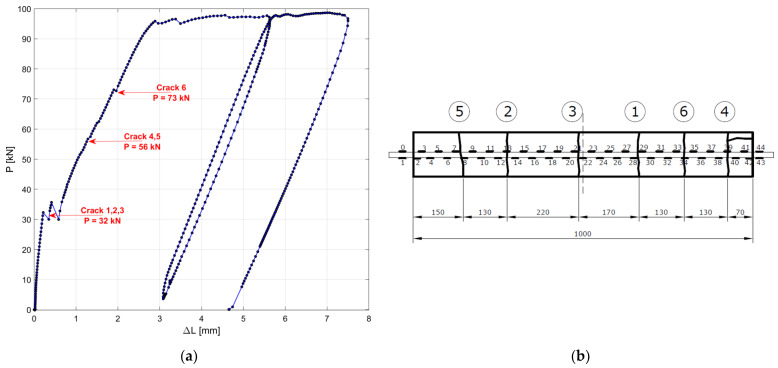
Experimental load–displacement diagram specimen n.1 (**a**), and location of the six cracks numbered according to the sequence of occurrence (unit: mm) (**b**).

The experimental strains along the steel rebar embedded in the r.c. tie are shown in Figure 11 for different levels of applied load *P*, before (a) and after crack openings (b), while in Figure 12 the experimental load elongation diagram (a) and the location of the cracks (b) are reported. The first three cracks (1, 2 and 3) opened under a load *P* approximately equal to 30 kN, while two subsequent cracks opened at *P* = 58 kN (4–5) and a last crack (6) opened at *P* = 74 kN. The load was increased till the yielding of the rebar, but no more cracks opened.

Looking at Figure 12a, it can be determined that, when a stabilized crack pattern is obtained, the slope of the loading cycle is nearly coincident with the slopes of the unloading and reloading cycles, even when irreversible slips occur.

**Figure 13:**
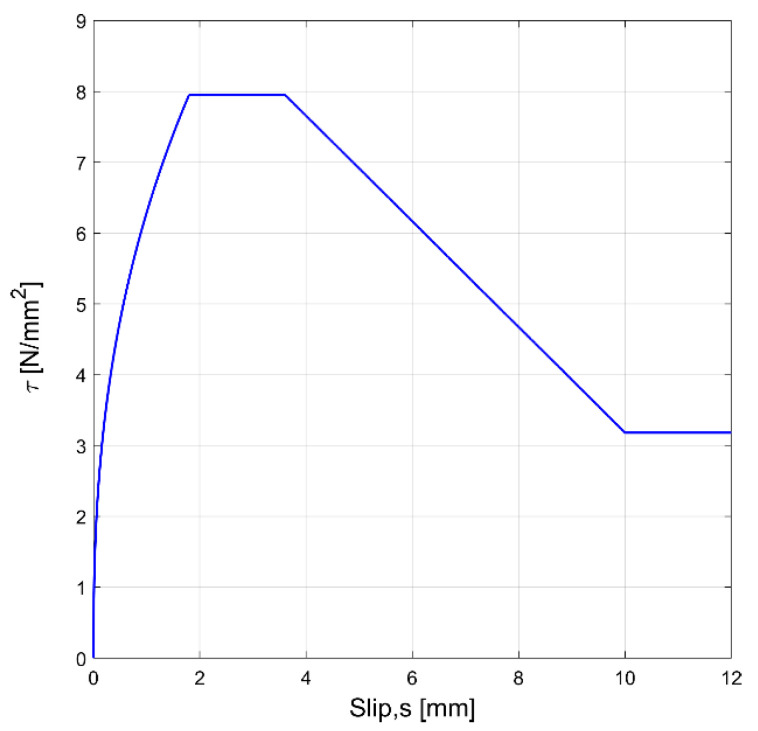
CEB-MC2010 bond stress-slip model, with $\tau_{b,\max}=1.25\sqrt{f_{cm}}=7.95\;N/{\mathrm{mm}}^{2}$, $\tau_{bf}=0.4\tau_{b,\max}$, α=0.4, $s_{1}=1.8\;\mathrm{mm}$, $s_{2}=3.6\;\mathrm{mm}$, and $s_{3}=10\;\mathrm{mm}$.

The experimental results were compared with those obtained by implementing the analytical model and the numerical procedure described in Section 2. In the following numerical analysis, the analytical bond stress–slip relationship defined in the CEB-FIB Model Code 2010 [26] is considered

(12) $$\tau \left(s\right)=\left\{\begin{array}{c} \tau_{b,\max}{\left(\frac{s}{s_{1}}\right)}^{\alpha }\;\;\;\;\;\;\;\;\mathrm{for}\;0\leq s\leq s_{1}\\ \tau_{b,\max}\;\;\;\;\;\;\;\;\mathrm{for}\;s_{1}\leq s\leq s_{2}\\ \begin{array}{c} \tau_{b,\max}-\left(\tau_{b,\max}-\tau_{bf}\right){\left(\frac{s-s_{2}}{s_{3}-s_{2}}\right)}^{\alpha }\;\;\mathrm{for}\;s_{2}\leq s\leq s_{3}\\ \tau_{bf}\;\;\;\;\;\;\;\;\mathrm{for}\;s>s_{4} \end{array} \end{array}\right.$$

where the parameters $\tau_{b,\max}$, $\tau_{bf}$, $s_{1}$, $s_{2}$, $s_{3}$, and α are defined in the Model Code as depending mainly on the failure mode—pull-out or splitting—and on the bond conditions within each failure mode. The FIB-MC2010 bond–slip model, which is derived for the investigated specimen, is illustrated in Figure 13. 

**Figure 14:**
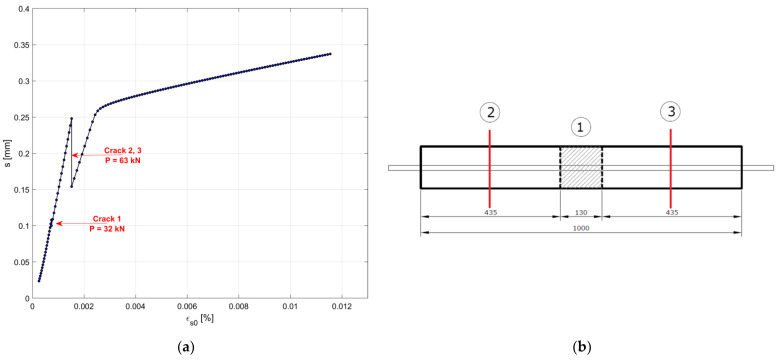
Analytical slip–strain diagram for specimen n.1 (**a**), and the location of the three cracks numbered according to the sequence of occurrence (unit: mm) (**b**).

The resulting slip–strain diagram, simulated for specimen n. 1, is shown in Figure 14. A first crack occurs at a distance from the end $L_{f}=435\;\mathrm{mm}$ when the applied load P reaches the value of 32.4 kN. When increasing the value of the load, a new crack is recorded in the two lateral portions of the specimen, delimited by the first crack, for P=62.9 kN. It is worth recalling that the loads inducing the cracks in the test were 30 kN, 58 kN and 74 kN respectively. Additionally, the crack spacing is quite different when comparing the numerical simulation and the experimental behavior of the same specimen. On average, the spacing observed during the test is around 300 mm, which is lower than the estimated $L_{f}$.

Furthermore, the analytical model, adopting the CEB-MC2010 bond–slip relationship, was able to predict only three cracks out of the six observed during the test.

Finally, it is observed that the magnitude of slip values recorded during the experimental campaign is very limited (<1 mm) with respect to those given by the CEB-MC2010 model. In fact, the sliding values that should be reached to activate the bond failure appear to be far too high and are never recorded during the tests.

**Figure 15:**
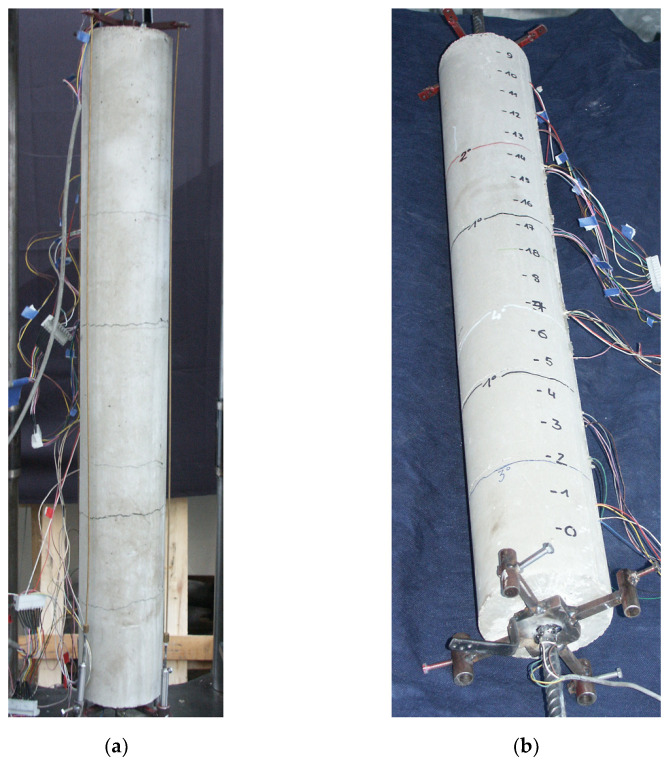
Reinforced concrete specimen no.5 tested at the Laboratory of the University of Pisa: (**a**) specimen during the test, (**b**) specimen after the test.

Similar conclusions can be drawn by checking the results from the remaining long specimens, where the cracks appeared in the same areas, and for similar slip values (see, for example, Figure 15, referring to specimen no. 5, before and after the test). The short specimen conception and results are described in the following section.

This experimental evidence indicates the need for a new model for the bond–slip behavior calibrated for the simulation of real structural members. Such a new bond–slip model for r.c. members will be presented and discussed in the next section. 

### 3.2. Short Specimen

**Figure 16:**
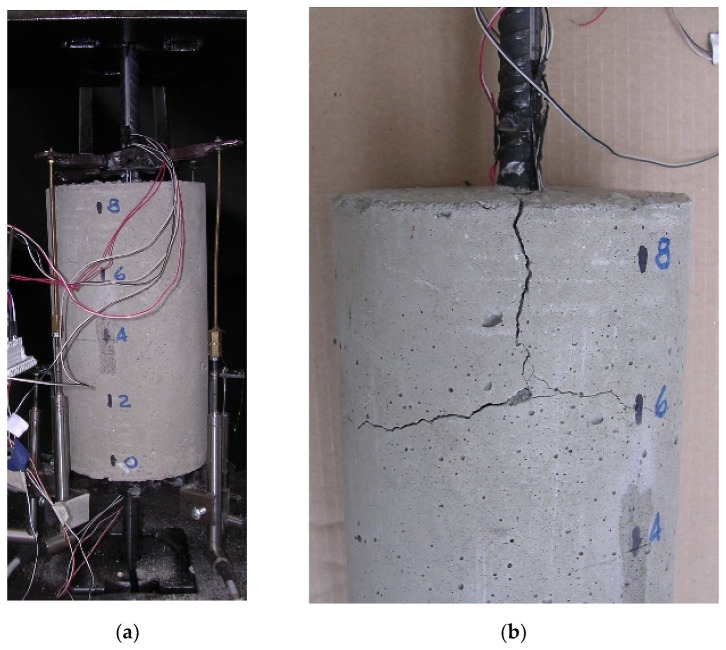
Reinforced concrete specimen n.3 tested at the Laboratory of the University of Pisa: (**a**) specimen before the test, (**b**) cracked specimen after the test.

An example of a “short” specimen (specimen no. 3 in Table 1) is illustrated in Figure 16, where cracks due to splitting were evident at the end of the test. It is important to highlight that cracks due to splitting were obtained for high values of the load close to the end of the test, thus not compromising the experimental test results on the bond–slip behavior of the short specimen.

**Figure 17:**
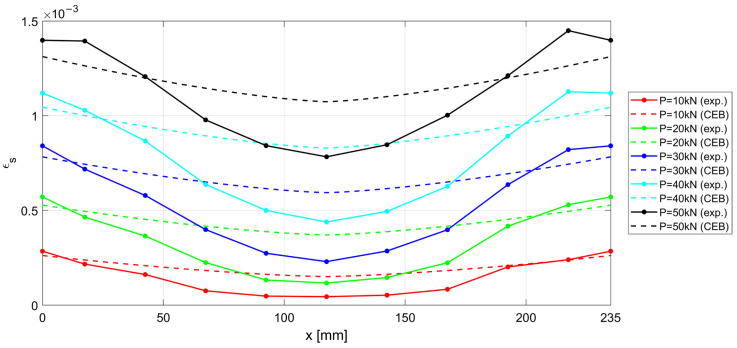
Experimental and numerical ε−x for increasing load P.

**Figure 18:**
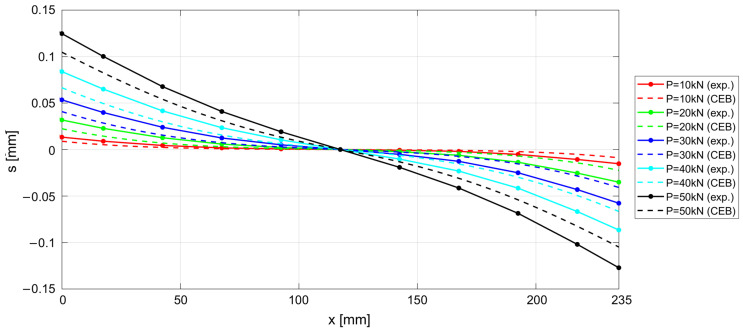
Experimental and numerical s−x for increasing load P.

**Figure 19:**
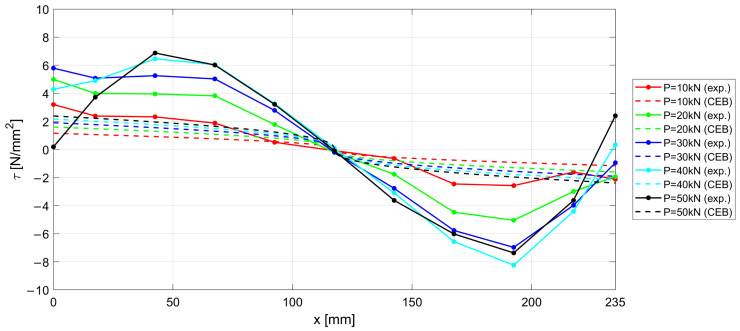
Experimental and numerical τ−x  curves for increasing load P.

The experimental results measured along the r.c. tie for different levels of the applied load *P* are shown in Figure 17, Figure 18 and Figure 19, considering the deformations on the steel rebar $\varepsilon_{s}$ (Figure 17), the slips s (Figure 18), and the variation in shear stress τ (Figure 19). In the same figures, the analytical curves obtained by implementing the numerical procedure described in Section 2 are reported. These curves were derived by adopting, as in the previous case, the CEB-MC2020 bond–slip law.

The comparison of experimental and numerical curves in Figure 17, Figure 18 and Figure 19 shows that the use of the CEB bond–slip model has the following effects:

- It leads to an underestimation of the bond stress transfer from steel to concrete. In fact, the steel deformations are characterized by a constant overestimation of the stresses in the bars, especially in the central section of the sample (see Figure 17), thus leading to concrete stresses significantly lower than in the real case;
- Nonetheless, a satisfactory level of approximation is obtained for the slip at the end sections of the r.c. tie, which estimates the crack width (see Figure 18), but the corresponding shear stresses are significantly different from the experimental ones. In fact, maximum slips, which are limited to 0.2 mm, are one order of magnitude lower than the slips associated with the plastic range of the bond–slip CEB model (see Figure 13). This outcome confirms that the actual magnitude of the slip values is well below that corresponding to the horizontal plateau or to the descending branch of the CEB bond–slip model.

From the above considerations, it is concluded that, using the CEB bond–slip model, long sections of confined bar are required to achieve cracking, due to the modest tangential stresses that are activated; consequently, the distance between the cracks increases, in a way that does not appear to be justified on the basis of engineering experience.

## 4. Discussion

As is highlighted in the Section 3, the results of the experimental campaign suggest the development of a new model for the bond–slip behavior of real r.c. members, especially to capture the local magnitude of slips, which are significantly lower than the theoretical ones provided by the CEB-MC2010 [26].

**Figure 20:**
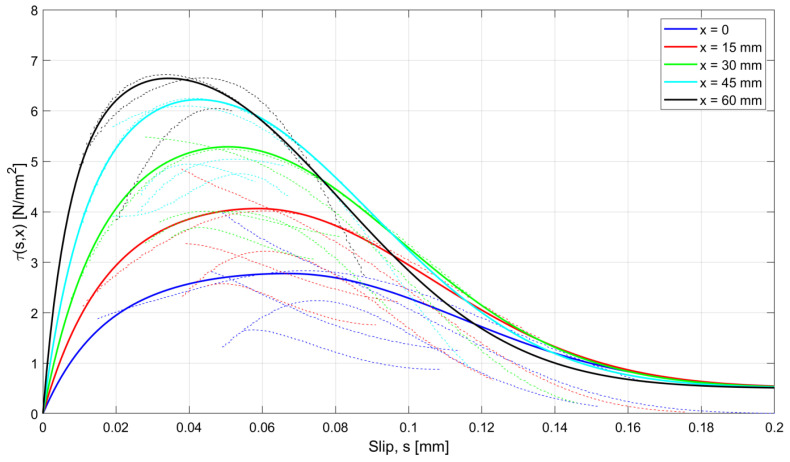
Comparison of the experimental bond–slip τ−s diagram, dashed lines; the proposed model for different abscissas *x* of the elements, solid lines.

The whole set of experimental results is represented in terms of bond–slip curves in Figure 20, where the experimental curves are drawn with dashed lines, parametrized according to values of the abscissa x varying between 0 and 60 mm, at 15 mm steps. In the diagram, the color of the curve depends on the value of the parameter x: the blue lines correspond to x=0; red lines to x=15 mm; green lines to x=30 mm; cyan lines to x=45 mm; black lines to x=60 mm.

To fit the experimental curves, a new model for bond–slip behavior has been developed and calibrated. Among the simple analytical models that have been considered and suitably calibrated to reproduce the experimental results, the one providing the most satisfactory predictions results an exponential model expressed by the equations:

(13) $$\tau \left(s,x\right)=\left\{\begin{array}{cc} \frac{\tau_{b,\max}\left(x\right)\left(e^{-b_{1}\left(x\right)s_{\max}^{2}\left(x\right)}-e^{-b_{1}\left(x\right){\left(s-s_{\max}\left(x\right)\right)}^{2}}\right)}{e^{-b_{1}\left(x\right)s_{\max}^{2}\left(x\right)}-1}\;\left[\mathrm{MPa}\right] & \;\mathrm{for}\;0\leq s\leq s_{\max}\\ \left(\tau_{b,\max}\left(x\right)-\tau_{bf}\right)e^{-b_{2}\left(x\right){\left(s-s_{\max}\left(x\right)\right)}^{2}}+\tau_{bf}\;\left[\mathrm{Mpa}\right] & \mathrm{for}\;s>s_{\max} \end{array}\right.$$

In the present study, the calibration provided the following values of the relevant model parameters:

(14) $$b_{1}=-16.06\;e^{0.074\;x}-350\;\left[{\mathrm{mm}}^{-2}\right]$$

(15) $$b_{2}=6.33\cdot 10^{-5}\cdot e^{0.171\;x}+222\;\left[{\mathrm{mm}}^{-2}\right]$$

(16) $$\tau_{b,\max}=-1.106\cdot 10^{-5}\cdot x^{3}+3.542\cdot 10^{-4}\cdot x^{2}+0.083\cdot x+2.778\;\left[\mathrm{Mpa}\right]$$

(17) $$s_{\max}=0.067-5.462\cdot 10^{-5}\cdot x\;\left[\mathrm{mm}\right]$$

where the abscissa x and the slip s are both expressed in mm. In fact, the reference bond stress–slip curves, represented by the solid lines in Figure 20, appear close enough to the experimental ones.

**Figure 21:**
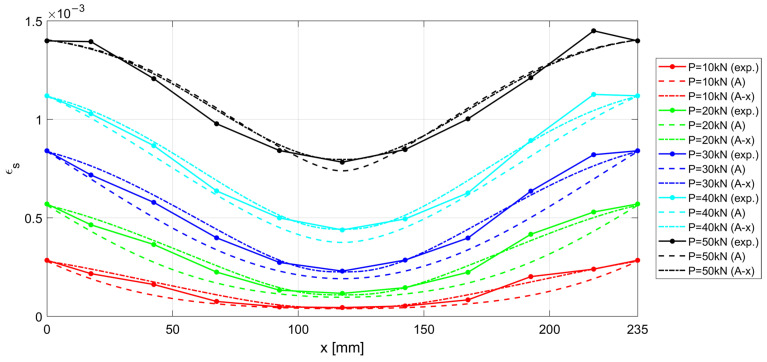
Experimental and numerical ε−x for increasing load P. Two numerical formulations are presented, obtained by considering a fixed bond–slip model (A) and a model with a varying abscissa (A-x).

**Figure 22:**
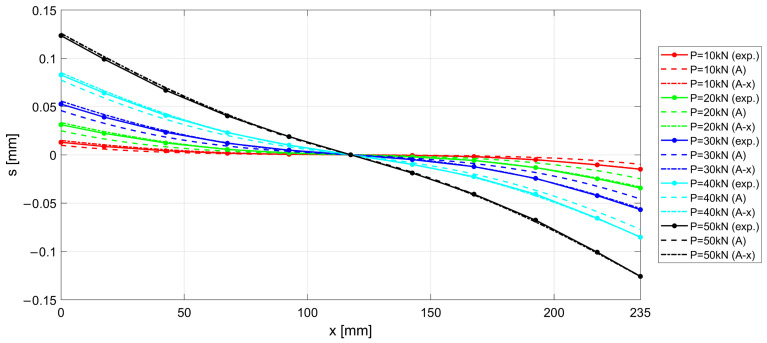
Experimental and numerical s−x for increasing load P. Two numerical formulations are presented, obtained by considering a fixed bond–slip model (A) and a model varying the abscissa (A-x).

**Figure 23:**
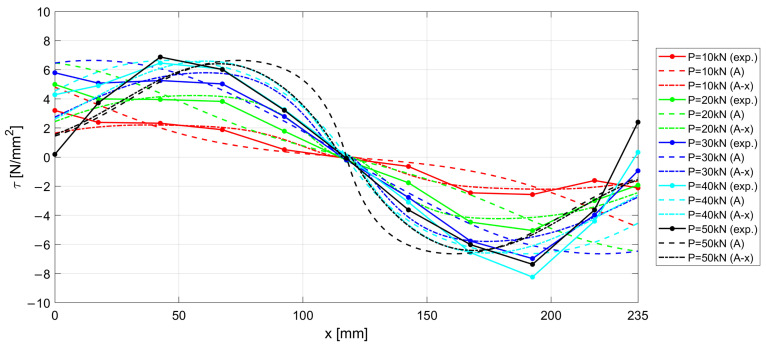
Experimental and numerical τ−x  curves for increasing load P. Two numerical formulations are presented, obtained by considering a fixed bond–slip model (A) and a model varying the abscissa (A-x).

When adopting the proposed bond stress–slip model, the analytical ε−x, s−x, and τ−x curves are derived by means of the numerical procedure introduced in Section 2, for the specimen investigated in Section 3.2, identified as n.3 in Table 1. The results are compared with the experimental curves in Figure 21, Figure 22 and Figure 23, for steel deformation $\varepsilon_{s}$, slip s, and shear stresses τ, respectively. In the figures, the analytical curves are shown that were obtained by considering the variation in the bond–slip model for different locations along the tie (point dashed lines), or a fixed bond–slip model (x=60 mm). 

When comparing the curves obtained by numerical integration adopting the proposed exponential bond–slip law (Figure 21, Figure 22 and Figure 23) with those previously obtained by using the CEB bond slip law (Figure 17, Figure 18 and Figure 19), it clearly emerges that the proposed model allows not only a better estimate of the actual crack widths, which are the ordinates at the end sections x=0 and x=235 mm, of the s−x diagrams in Figure 18 and Figure 22, respectively, but also a more precise fitting of the experimental local behavior.

## 5. Conclusions

In this paper, the outcomes of an experimental campaign aiming to evaluate the local bond–slip relationship of rebars in r.c. members are presented and discussed. 

The innovative testing arrangement proposed consists of a r.c. tie, in which the steel rebar, suitably prepared, is fully instrumented with strain gauges placed at 25 mm intervals, in alternate positions, allowing one to derive the variation in the bond along the element as a function of the applied load. A refined local bond–slip relationship is thus obtained that can elaborate on the experimental data, thus overcoming the limitation of the standardized bond–slip law. 

The implementation of the obtained local bond–slip law in a numerical procedure allows one to reproduce the experimental behavior of the r.c. element under tension, not only at the ends or near the cracks, but also locally, independently of the considered abscissa.

The wide numerical study, presented in Section 3, demonstrates that the bond–slip law proposed by the CEB-FIP Model Code, although able to provide a satisfactory estimate of the crack width, which is the most relevant information from the design point of view, cannot be adopted for refined studies when a precise assessment of the local behavior is required. In this latter case, the original relationship proposed in the paper appears to be more appropriate.

## Figures and Tables

**Table 1:** Main characteristics of test specimen: length L, steel rebar diameter Φ, r.c. tie diameter $\Phi_{c}$, number of electrical strain gauges on the naked bar $N_{1}$ and on the embedded bar $N_{2}$, number of LVDT-A $N_{\mathrm{LVDT}-A}$, and of LVDT-B $N_{\mathrm{LVDT}-B}$.

N.	L (mm)	*Φ*^1^ (mm)	*Φ*_c_ (mm)	*N*_1_	*N*_2_	*N*_LVDT-A_	*N*_LVDT-B_
1	1000	16	132	4	40	4	4
2	235	16	132	1	9	4	2
3	235	16	132	1	9	4	2
4	235	16	132	1	9	4	2
5	1000	20	132	4	40	4	2
6	1000	20	132	4	40	4	-

^1^ Notional diameter of the ribbed bar.

**Table 2:** Results of compression tests on concrete specimens.

N.	*H* (mm)	*D* (mm)	Mass (kg)	Density (kg/m^3^)	*R* (kN)	*f_c_* (N/mm^2^)
1	195	100	3.45	2253	296	37.7
	195	100	3.45	2253	318	40.5
	195	100	3.42	2233	328	41.8
	195	100	3.44	2246	330	42.0
2, 3, 4	193	100	3.36	2217	320	40.7
	196	100	3.40	2209	311	39.6
5	196	100	3.52	2287	254	32.3
	196	100	3.52	2287	299	38.1
6	198	100	3.56	2289	234	29.8
	196	100	3.54	2300	236	30.0

**Table 3:** Results of indirect tensile tests on concrete specimens.

N.	*H* (mm)	*D* (mm)	Mass (kg)	Density (kg/m^3^)	*R* (kN)	*f_ct,sp_* (N/mm^2^)
1	200	100	3.54	2254	96	3.1
	200	100	3.54	2254	96	3.1
2, 3, 4	200	100	3.44	2190	101	3.2
	200	100	3.50	2228	91	2.9
5	200	100	3.56	2266	106	3.4
	200	100	3.56	2266	100	3.2
6	200	100	3.56	2266	105	3.3
	200	100	3.54	2254	125	4.0

**Table 4:** Results of direct uniaxial tensile tests on concrete specimens.

N.	*Φ*_c_ (mm)	*f_ct_* (N/mm^2^)	*E_ct_* (N/mm^2^)	*f_ctm_*(EC2) (N/mm^2^)	*E_ct_*(EC2) (N/mm^2^)
1	132	2.32	29,000	3.5	35,277
2	132	2.16	24,100	2.4	30,835
3	132	2.05	21,200	3.1	33,468

## Data Availability

The data presented in this study are available on request from the corresponding author. The data are not publicly available as they cannot be used for commercial purposes.
